# Model systems informing mechanisms and drug discovery: a review of *POLG*-related disease models

**DOI:** 10.12688/wellcomeopenres.18637.2

**Published:** 2025-04-04

**Authors:** Jonathan Meyrick, Renae J Stefanetti, Linda Errington, Robert McFarland, Gráinne S. Gorman, Nichola Z. Lax

**Affiliations:** 1 Wellcome Centre for Mitochondrial Research, Faculty of Medical Sciences, Newcastle University, UK, Newcastle upon Tyne, NE24HH, UK; 2 NIHR Newcastle Biomedical Research Centre, Newcastle University, UK, Newcastle upon Tyne, NE24HH, UK; 3 NHS Highly Specialised Service for Rare Mitochondrial Disorders, Newcastle upon Tyne Hospitals NHS Foundation Trust, Newcastle upon Tyne, NE24HH, UK; 4 Faculty of Medical Sciences, Newcastle University, UK, Newcastle upon Tyne, NE24HH, UK

**Keywords:** POLG, mtDNA, epilepsy, mitochondria, neurological manifestations, preclinical

## Abstract

**Introduction:**

Pathogenic variants in the gene encoding the catalytic subunit of DNA polymerase gamma ( *POLG*), comprise an important single-gene cause of inherited mitochondrial disorders. Clinical manifestations are now recognised as an array of overlapping clinical features rather than discrete syndromes as originally conceptualised. Animal and cellular models have been used to address numerous scientific questions, from basic science to the development and assessment of novel therapies. Here, we sought to employ systematic approaches, wherever possible, to investigate the cellular and animal models used in *POLG*-related research and assess how well they help us understand disease mechanisms in patients.

**Methods:**

Four databases were searched from inception to May 31 ^st^, 2022: MEDLINE, Scopus, Web of Science, and Cochrane Review. Original articles available in English, reporting the use of a model system designed to recapitulate *POLG*-related disease, or related pathogenicity, were eligible for inclusion. Risk of bias and the methodological quality of articles were assessed by an adapted version of the Cochrane Risk of Bias Tool, with the quality of evidence synthesized across each model.

**Results:**

A total of 55 articles, including seven model organisms (Human, yeast [ *Saccharomyces cerevisiae* and *Schizosaccharomyces pombe*], *Drosophila*, Mouse, *Caenorhabditis elegans*, and Zebrafish) with 258 distinct variants were included. Of these, 69% (N=38/55) of articles recapitulated mitochondrial DNA (mtDNA) depletion, 33% (N=18/55) utilised tissue-specific models of *POLG*-related dysfunction, while 13% (N=7/55) investigated the effect of potential therapeutics in *POLG*-related mitochondrial disorders.

**Discussion:**

While some evidence is available to support the ability of *POLG*-related disease models to recapitulate molecular mechanisms and phenotypes, much is of limited quality, with inconsistencies evident across the literature. Further success in examining and translating novel therapies into effective treatments will be enhanced by the availability of more robust models that better recapitulate the entire spectrum of *POLG*-related disease.

**PROSPERO registration:**

CRD42021234883

## Abbreviations

AHS, Alpers–Huttenlocher Syndrome; CLO, clofilium tosylate; EHNA, Erythro-9-(2-hydroxy-3-nonyl) adenine; iPSC, induced-pluripotent stem cell; PEO, Progressive External Ophthalmoplegia; PRISMA, Preferred Reporting Items for Systematic Reviews and Meta-Analyses; qPCR, quantitative polymerase chain reaction.

## Introduction

The nuclear gene *POLG* encodes for the catalytic subunit of the mitochondrial DNA polymerase gamma (pol γ), the enzyme that replicates mitochondrial DNA (mtDNA) ^1^. *POLG* variants are reported to be the most common cause of inherited mitochondrial disorders; characterized by mtDNA deletions or depletion (or both) in symptomatic tissues ^2^. The clinical spectrum of *POLG-*related disease has historically been categorised into six major syndromes ^3^. However, it is now recognised that phenotypically, *POLG*-related disorders and their clinical manifestations, clearly form a continuum, necessitating a new, simplified approach to its classification ^4^.

While there have been significant advancements in our understanding of mitochondrial disease genetics and diagnosis in recent years, there are currently no disease-modifying therapies available for *POLG*-related diseases ^3^. Preclinical models are emerging as promising candidates, however a comprehensive evaluation of the effectiveness of these models, in the context of *POLG*-related mitochondrial disease, has yet to be robustly performed.

In order to understand the molecular mechanisms underlying *POLG*-related disease and inform future therapeutic targets, researchers have attempted to create models that recapitulate human disease, but evidence that these models accurately reflect human *POLG*-related disease is lacking. To close this knowledge gap, we sought to investigate the effectiveness of cell and animal models to recapitulate features of *POLG*-related disease, from a molecular, genetic, and phenotypic perspective, using a systematic approach wherever possible.

## Methods

### Protocol registration

This review was conducted in accordance with Preferred Reporting Items for Systematic Reviews and Meta-Analyses (PRISMA) guidelines (PRISMA checklist available in Supplementary Material, Table 1 [Extended data ^5^]). The protocol was prospectively registered in the PROSPERO International Prospective Register of Systematic Reviews (registration ID: CRD42021234883).

### Deviations from Protocol

During development of the review, several substantial deviations were made from the original PROSPERO-registered protocol. These deviations are listed here, along with their rationale:

*Review question –* The registered PROSPERO protocol initially aimed to address four research questions. However, this review is the focus of a single question (Question 3: *What are the molecular genetic mechanisms underpinning POLG-related disorders*). To enhance clarity, this question was rephrased to specifically investigate the effectiveness of cell and animal models recapitulating molecular, genetic, and phenotypic features of *POLG*-related disease.

*Type and method of review –* This original protocol outlines a systematic review. However, given the nature and scope of the available literature, it was appropriate to deviate from the approach. Instead, the current review encompassed a scoping review while employing systematic elements wherever possible, including a systematic literature search and a risk of bias assessment.

*Eligibility criteria –*

Inclusion criteria: The registered protocol included human-based studies as an inclusion criterion. However, in accordance with the revised research question, only studies utilising animal and/or cellular models were included. Therefore, human-based studies were removed from the inclusion criteria.

Exclusion criteria: Not included in the registered protocol, studies utilising the polg-D257A ‘mutator’ mouse model were excluded. During the literature screening phase, thorough discussion among investigators determined that this model does not accurately model does not reflect clinical *POLG*-related disease. Therefore, studies utilising the polg-D257A ‘mutator’ mouse model were excluded to prevent misrepresentation of the animal and cellular models of clinical *POLG*-related disease. The eligibility criteria applied in this review are detailed in the Supplementary Material, Table 3 [Extended data ^5^].

*Data extraction and synthesis –*

The registered protocol included data extraction of human-based studies. In line with the revised research question, data were extracted exclusively from studies including cellular/animal models.

Furthermore, the registered protocol did not detail how the effectiveness of different models would be assessed. To address this, molecular indications (e.g., mtDNA levels) and exhibited phenotypes (e.g., myopathy) were evaluated across included studies (see Supplementary Material, Table 3 [ *Extended data* ^5^]). Any affected measures were reported for each included article (see Supplementary Material, Table 5 [ *Extended data* ^5^]) and subsequently aggregated across articles to establish a majority-based consensus on the extent to which these measures recapitulated features of *POLG*-related disease ( Table 2).

### Search strategy

We searched Medline, SCOPUS, Web of Science, and the Cochrane Library for articles published from inception to 31 ^st^ May, 2022, with subsequent language restrictions applied (see Supplementary Material, Table 2 [ *Extended data* ^5^]). We performed backward citation searching, and hand searching to manually screen the reference lists of included articles and related reviews. The search was overseen by a senior medical librarian (L.E.), and peer reviewed by the investigational team whilst piloting the search strategy, and prior to final execution. Records were imported using EndNote 20x bibliographic management software for de-duplicating, screening and managing the eligibility process. The search strategy applied, was translated as closely as possible across databases with no search filters applied for comprehensiveness.

### Eligibility criteria

The following inclusion criteria were applied: (i) use of animal or cellular model system(s) designed to recapitulate putative *POLG*-related disease, from a molecular, genetic, and/or phenotypic perspective ^6^; use of standardised measures to assess model recapitulation; and (iii) published journal articles, notes or short communications available in the English language. No restriction was placed on publication date or study design, to increase comprehensiveness. Articles relating to the polg-D257A ‘mutator’ mouse model were excluded, as it was deemed this model does not truly recapitulate *POLG*-related mitochondrial disease (see full eligibility criteria in Supplementary Material, Table 3 [ *Extended data* ^5^]) ^7^.

### Study selection

Three authors (J.J.M., N.Z.L., and R.J.S.) independently screened all records by titles and abstracts for eligibility and five authors (J.J.M., N.Z.L., R.J.S., G.S.G. and R.M.) assessed the full texts of potentially eligible articles to determine qualification for final inclusion. Conflicts on inclusion of articles were resolved by consensus through discussion. Reason for exclusion of full text records is provided (Supplementary Material, Table 4 [ *Extended data* ^5^]).

### Data extraction

Data extraction from included articles was performed independently by J.M. and N.Z.L and accuracy checked by all other investigators. Data extracted included methodology of models generated and molecular mechanisms (quantification of mtDNA maintenance defect; quantitative measures of mtDNA deletion levels and copy number, histological or biochemical data); phenotypic/clinical recapitulation data; sex and age-related data; and conclusion surrounding model efficacy (see Supplementary Material, Table 5 [ *Extended data* ^5^]). The degree of each model’s effectiveness was determined by the molecular indications, such as mtDNA levels, or exhibited phenotypes such as myopathy (see Supplementary Material, Table 3 [ *Extended data* ^5^]). Any affected measures were then reported for each included article (see Supplementary Material, Table 5 [ *Extended data* ^5^]).

### Risk of bias / summary of evidence

Risk of bias was assessed independently by two investigators (J.J.M. and R.J.S.) using an adapted version of the Cochrane Risk of Bias Tool (see Supplementary Material, Table 6A [ *Extended data* ^5^]) ^8^. Articles were individually rated as having a low, high, or unclear risk of bias according to meeting pre-defined criteria, including the reporting quality and standardisation of methods (see Supplementary Material, Tables 6A–6B [ *Extended data* ^5^]). The overall quality of appraisal and summary of evidence across each model was then synthesized based on the majority of evidence for each model ( Table 2).

### Statistical analysis

The methodological quality of the included studies was limited, with variability of extracted data precluding a meta-analysis. Compatible results across model systems, such as the presentation of common phenotypes or quantifiable levels of mtDNA depletion, were aggregated across articles for interpretation.

### Data availability

All data underlying this review (including raw data extracted, articles reviewed and bias assessment for individual articles) have been made available in a publicly accessible data repository ( Figshare [ *Underlying data* ^5^]).

## Results

### Overview

**Figure 1.**
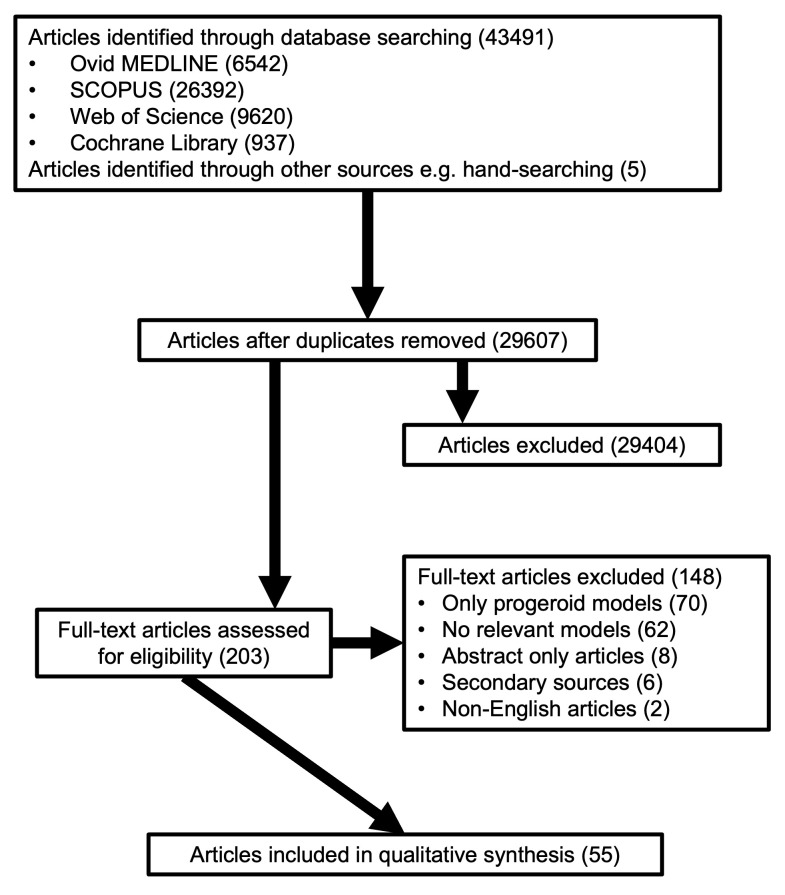
PRISMA Flow Diagram. The PRISMA flowchart depicts the article selection process, with a total of 55 articles included for final review.

The screening and selection of articles are described in Figure 1 (also see Supplementary Material, Figure 1 [ *Extended data* ^5^]). Of the 29,607 articles, 55 articles met the selection criteria. To ensure the fidelity of data analysed, duplicate model data (N=1/55 articles) were removed from the analysis.

Seven different model systems were used across all included articles (N=55). This included categorisation by model type: human-derived (N=24/55 [44%], with 9% (N=5/55) of these using induced-pluripotent stem cell (iPSC)-derived); *Saccharomyces cerevisiae (S. cerevisiae)* yeast (N=19/55 [35%]); *Drosophila melanogaster* flies (N=6/55 [11%]); *Mus musculus* mouse (N=5/55 [9%]; *Caenorhabditis elegans* nematodes (N=2/55 [4%]); *Danio rerio* zebrafish (N=2/55 [4%]); and *Schizosaccharomyces pombe (S. pombe)* yeast (N=1/55 [2%]).

The effectiveness of the reviewed models, as determined by the levels of mtDNA depletion or exhibited phenotype, was highly variable ( Table 1). Of all included articles (N=38/55), 69% reported model systems that were able to demonstrate a significant depletion of mtDNA, indicative of aberrant pol γ or its homologs, and impaired mtDNA replication; clearly affecting mitochondrial function. Additionally, 42% (N=23/55) of included articles demonstrated an ability to recapitulate a phenotypic form of impaired mitochondrial function, albeit limited, such as a very particular form of hepatic fibrosis in cases of Alpers-Huttenlocher syndrome (AHS) ^3^.

**Table 1.** Summary and demographics.

	*Human* *(iPSC-derived* *only)*	*Human (all)*	*S. cerevisiae* *yeast*	*S. pombe* *yeast*	*C. elegans ^i^*	*Zebrafish*	*Mouse*	*Drosophila*	*Total ^h^*
***Total (N, articles) (n,*** ***institutes) ^a^***	**N=5 (9%); n = 1**	**N=24 (44%);** **n = 17**	**N=19 (35%);** **n = 12**	**N=1 (2%); n = 1**	**N=2 (4%); n = 2**	**N=2 (4%); n = 2**	**N=5 (9%); n = 3**	**N=6 (11%); n = 4**	**N=55; n = 36**
*Multiple organism*	Nil	N=3	N=3	Nil	N=1	Nil	Nil	Nil	N=3
***Sex, female/*** ***male (n) (%)*** ***Unknown (n)***	**5/5; 50/50%** **0 (0%)**	**12/29; 12/29** **% 60 (59%)**	**N/A**	**N/A**	**N/A**	**2/2; 28/28%** **3 (44%)**	**1/1; 17/17%** **4 (66%)**	**15/14; 52/48%** **0 (0%)**	**35/51; 23/34% 67** **(43%)**
*Sex, herma-phroditic/male (n) (%)* *Unknown (n)*	**N/A**	**N/A**	**N/A**	**N/A**	**1/1; 50/50%** **0 (0%)**	**N/A**	**N/A**	**N/A**	**1/1; 50/50%** **0 (0%)**
***Phenotype ^b^***	**N/A**	**N/A**	**N/A**	**N/A**	**N=1**	**Nil**	**N=3**	**N=4**	**N=8 (15%)**
*Fibrosis*	N/A	N/A	N/A	N/A	Nil	Nil	N=1	Nil	N=1
*Myopathy*	N/A	N/A	N/A	N/A	Nil	Nil	N=1	Nil	N=1
*Necrosis*	N/A	N/A	N/A	N/A	Nil	Nil	N=1	Nil	N=1
*Growth arrest ^c^*	N/A	N/A	Nil	Nil	N=1	Nil	Nil	N=4; n=10	N=5; n=11
***Tissue Specific Models***	**N=5; n=4**	**N=12; n=5**	***N/A***	***N/A***	***Nil***	**N=1; n =1**	**N=3; n=4**	**N=3; n=3**	**N=18 (33%); n=5**
Skeletal muscle	Nil	N=6	N/A	N/A	Nil	Nil	N=1	N=1	N=8
Neurons	N=3	N=3	N/A	N/A	Nil	Nil	Nil	N=1	N=4
Cardiac	N=1	N=1	N/A	N/A	Nil	Nil	N=3	Nil	N=4
Hepatic	N=2	N=7	N/A	N/A	Nil	Nil	N=1	Nil	N=8
Central nervous system	N=1	N=2	N/A	N/A	Nil	N=1	N=1	N=1	N =4
***Genotypes analysed (n,*** ***unique)***	**N=9; n=3**	**N=84; n=66**	**N=147;** **n=105**	**N=1; n=1**	**N=3; n=2**	**N=7; n=7**	**N=6; n=5**	**N=20; n=14**	**N=258; n=200**
*POLG A467T ^d^*	N=5; n=2	N=14; n=17	N=1; n=1	Nil	Nil	Nil	N=1; n=1	Nil	N=16; n=19
*POLG W748S ^d^*	N=4; n=2	N=11; n=12	N/A	N/A	Nil	Nil	Nil	Nil	N=11; n=12
***Model Specific Endpoints***	**N/A**	**N/A**	**N=28; n=208**	**N/A**	**N/A**	**N/A**	**N/A**	**N/A**	**N=28; n=208**
*Petite colony formation ^e^*	N/A	N/A	N=15; n=83	N/A	N/A	N/A	N/A	N/A	N=15; n=83
*Erythromycin resistant* *colony Formation ^f^*	N/A	N/A	N=13; n=125	N/A	N/A	N/A	N/A	N/A	N=13; n=125
***mtDNA depletion ^g^***	**N=3; n=2**	**N=21; n=99**	**N=3; n=18**	**N=1; n=1**	**N=2; n=2**	**N=2; n=1**	**N=5; n=9**	**N=4; n=10**	**N=38 (69%); n=140**
*≤ 49%*	N=2, n=2	N=10, n=25	N=3; n=7	Nil	Nil	Nil	N=4; n=7	N=3; n=5	N=20; n=43
*50 ≤ x < 75%*	N=2; n=3	N=9; n=24	N=2; n=7	Nil	Nil	Nil	N=1; n=1	N=2; n=4	N=14; n=36
*75 ≤ x 99%*	N=1; n=1	N=10; n=45	N=1; n=2	Nil	N=2; n=3	N=1; n=1	N=1; n=1	N=1; n=1	N=16; n=53
*x ≥ 99%*	Nil	N=2; n=5	N=1; n=2	N=1; n=1	N=1; n=1	Nil	Nil	Nil	N=5, n=9
***Thera-peutics analysed*** ***(n, total thera-peutics)***	**N=1; n=2**	**N=3; n=4**	**N=1; n=1**	**Nil**	**N=1; n=1**	**N=1; n=1**	**N=1; n=1**	**N=1; n=1**	**N=7 (13%); n=6**
*CLO*	Nil	N=1, n=1, x=1	N=1, n=11, x=11	Nil	N=1, n=1, x=1	N=1, n=2, x=2	Nil	Nil	N=2, n=15, x=15
*Nicotin-amide riboside*	N=1, n=2, x=0	N=1, n=2, x=0	Nil	Nil	Nil	Nil	Nil	Nil	N=1, n=2, x=0
*Metformin*	N=1, n=2, x=0	N=1, n=2, x=0	Nil	Nil	Nil	Nil	Nil	Nil	N=1, n=2, x=0
*Zidovudine (AZT)*	Nil	Nil	Nil	Nil	Nil	Nil	N=1, n=1, x=0	Nil	N=1, n=1, x=0
*AOX*	Nil	Nil	Nil	Nil	Nil	Nil	Nil	N=1, n=1, x=0	N=1, n=1, x=0
*EHNA*	Nil	N=1, n=5, x=5	Nil	Nil	Nil	Nil	Nil	Nil	N=1, n=5, x=5

Data is N = number of articles; n = number of models (unless otherwise stated); x = affected models Summary data includes the utilising specific model types, and quantification details of; clinical phenotype recapitulation, tissue analysis, treatment assessment. Abbreviations: AOX = Alternative oxidase; CLO = Clofilium tosylate; EHNA = Erythro-9-(2-hydroxy-3-nonyl) adenine ^a^ number of distinct research institutes listed as the primary institute of research within an article. ^b^ recapitulation of any phenotypes observed in clinical *POLG*-related disease. ^c^ described as larval arrest in *Caenorhabditis elegans nematodes* and a lack of larval or pupal viability in *Drosophila melanogaster* ^21^. ^d^ homo- or heterozygous, including equivalent variants in model organism. ^e^ formation of petite colonies in *Saccharomyces cerevisiae* is well established as an indicator of mitochondrial dysfunction ^22^ ^f^ formation of erythromycin resistant colonies in *Saccharomyces cerevisiae* is well established as an indicator of mitochondrial dysfunction ^22^. ^g^ mtDNA copy number is an indication of polymerase activity, with a decrease reflecting defective mtDNA replication due to impaired polymerase activity ^23^. ^h^ Supplementary Material, Table 5 [ *Extended data* ^5^] provides details relating to model system utilisation across for each included article.

**Table 2.** Summary of Evidence across models ^a^.

Domains of Model Effectiveness: Was the model?	Human (iPSC-derived only) ^b^	Human (all)	S. cerevisiae yeast	S. pombe yeast	C. elegans	Zebrafish	Mouse	Drosophila
Effective in recapitulating *POLG*-related disease genetics	Yes	Yes	Yes	No	Unclear	No	Yes	Yes
Effective in recapitulating mtDNA depletion	Yes	Yes	Unclear	Yes	Yes	Yes	Unclear	Unclear
Effective in recapitulating tissue-specific *POLG*-related disease	Yes	Yes	No	No	No	Yes	Yes	Yes
Effective in recapitulating *POLG*-related phenotypes	Unclear	No	Yes	No	Unclear	No	Yes	Unclear
Effective investigating potential treatment options	Unclear	Yes	Yes	N/A	Yes	Yes	Unclear	Unclear
**Risk of Bias Domains**								
Selective reporting	Low	Unclear	Unclear	Unclear	Unclear	Unclear	Low	Unclear
Consistent model generation	Unclear	Low	Unclear	Low	Unclear	Unclear	Low	Unclear
mtDNA scoring/ measurement	Low	Low	Unclear	Low	Unclear	Low	Low	Low
Model selection consistency	Low	Low	Unclear	Low	High	Unclear	Low	Unclear
Analysis method consistency	Low	Low	Low	Low	Low	Low	Low	Low

The effectiveness of each of the model system was summarised based on data synthesised across included articles (Supplementary Material, Table 5 [ *Extended data* ^5^]). Models were deemed as Yes/Unclear/No/Not applicable (N/A) in relation to their effectiveness to meet pre-defined criteria. Risk of bias for each model was deemed as Low/Unclear/High risk. Individual article appraisal is available (Supplementary Material, Table 6A–B [ *Extended data* ^5^]). ^a^ Supplementary Material, Table 5 [ *Extended data* ^5^] provides details relating to model system utilisation across for each included article. ^b^ All investigators agreed that it was important to consider iPSC models separately, based on their emergence as a distinct disease-specific cellular model in bioresearch ^24^.

While fibroblasts were used in 18 of the 24 human cellular-based articles (75%), more recent studies have utilised iPSC-derived cell types ^9– 13^, representing a shift towards iPSC-derived models, using retroviral reprogrammed fibroblasts. These articles successfully produced mutant cell types such as dopaminergic neurons and hepatocytes, with significant mtDNA depletion found across iPSC-derived cells. Additionally, Chen *et al.* demonstrated the ability of nicotinamide riboside and metformin to ameliorate mitochondrial dysfunction related to *POLG* variants, via changes in metabolic signal transduction and increased mitophagic turnover ^9, 14^.

### Molecular recapitulation

Of the included articles using *S. cerevisiae,* 27% (N=15/55) were able to generate ‘petite’ yeast colonies as a result of mtDNA depletion due to impaired polymerase activity ^15^. This direct morphological phenotype was confirmed with the orthogonal use of qPCR mtDNA measurement in 20% (N=3/15) of the 15 articles. Additionally, 24% (N=13/55) of articles indirectly assessed mtDNA depletion through mitoribosome defects and resistance to erythromycin.

The success of *S. cerevisiae* yeast models reflects the model’s simplicity and its use in large-scale studies. For example, Stumpf *et al.* (2010) ^16^ was able to assess the effects of 32 different *MIP1* variants on petite colony formation, and 18 mutants for mtDNA copy number. In this study, across all heteroallelic mutants, there was a mean mtDNA copy number fold-change of 15.8, when compared to wild-type strains, while monoallelic mutants possessed a mean fold-change of 10.2. Additionally, 44% of the mutants analysed were able to produce 100% petite colonies, indicating high levels of mtDNA depletion.

mtDNA levels in zebrafish were quantified via qPCR. It was ultimately determined that heterozygous zebrafish mutants showed no significant depletion of mtDNA, while homozygous mutants exhibited mtDNA depletion from an early stage.

*Drosophila* were seldom used, with most articles utilising alterations in *tamas* expression (that is, the mitochondrial DNA polymerase catalytic subunit gene in *Drosophila*). Martinez-Azorin *et al.* (2008, 2013) ^17, 18^ showed that *Drosophila* overexpressing *tamas* due to the GAL4 system, resulted in varied mtDNA depletion ranging from 40–70%, assessed by mtDNA:nuclear DNA (nDNA) ratio.

mtDNA copy number in *C. elegans* was assessed, with control worms’ mtDNA levels increased during their life cycle, while mutant worms did not demonstrate similar findings. Pitayu *et al.* (2016) ^19^ successfully demonstrated a potential treatment effect in nematodes using clofilium tosylate (CLO), an anti-arrhythmic agent targeting K ^+^ ion channels ^20^, with increased mtDNA content levels in mutant worms by approximately two-fold. However, the effect of CLO did not rescue the mtDNA depletion in mutant worms to the wild-type level.

Standardisation of methods was high across human cellular-based articles, with mtDNA quantification via qPCR used in 88% (N=21/24). This was used to establish an mtDNA:nDNA ratio, with 57% (N=12/21) of human cellular-based articles demonstrating a mtDNA depletion of >75%.

### Genetic significance

Levels of homology with human *POLG* varied among non-human models. While murine models possessed *POLG* exon homology of up to 95%, *S. cerevisiae yeast* models possessed only 43% sequence homology ^25^. The *Drosophila* homolog of pol γ, *tamas*, also possesses conserved motifs ^26^.

Models such as zebrafish were created through Transcription Activator-Like Effector Nuclease (TALEN) vectors, resulting in entire coding sequences removed. *C. elegans* models (ok1548) similarly had the removal of whole exons, pol γ exons 8–10, encoding part of the polymerase domain. Additionally, Bratic *et al.* (2010) ^27^ also used a model (tm2685) which has the first two exons of the exonuclease domain removed.

The use of patient-derived models provides a clear clinical relevance, given that diagnoses and mutational analysis can be conducted to demonstrate phenotype-genotype relationships. Of these articles, 76% reported pathogenic variants analysed in cellular models, while 60% occurred in clinical *POLG*-related disease, including prevalent *POLG* variants such as *p.Ala467Thr* and *p.Trp748Ser*, identified in AHS, Ataxia Neuropathy Spectrum and PEO.

### Phenotypic recapitulation

Mouse models were the most successful for phenotype recapitulation, with 60% of the articles that described murine models able to induce a phenotype. Cardiac phenotypes as a result of the transgenic pol γ p.Tyr955Cys variant were especially noteworthy, with a consistent phenotype produced by mutants selectively expressed in the heart via a cardiac specific α-myosin heavy chain promoter. These variants were able to induce cardiomyopathy and cardiac fibrosis, as determined by Lewis *et al.* (2007) ^28^ and Koczor *et al.* (2013) ^29^, respectively.

Only two publications utilised zebrafish models ^30, 31^, successfully generating zebrafish with miscoded sequences resulting in premature stop-codons within the zebrafish *POLG.* Rahn *et al.* (2015) ^31^ demonstrated that in a knockout of pol γ, zebrafish were able to survive beyond the juvenile stage, albeit with severe mtDNA depletion.

*Drosophila* studies were interesting as *tamas* overexpression was targeted to the nervous system and skeletal muscle cells and resulted in increased levels of pupal lethality in flies. Additionally, Rodrigues *et al.* (2018) ^32^ and Siibak *et al.* (2017) ^6^ both investigated single amino acid variants of *tamas*; Q1009A, and Y873H and Y873C, respectively. In all of these mutant flies, lethality was exhibited, with homo- or heterozygous Q1009A flies unable to live beyond the L3 larval stage. Meanwhile, variants to Y873, the *Drosophila* homolog of human *POLG* Y955, led to reduced affinity of the polymerase for mtDNA. In homozygous Y873H and Y873C mutants, mtDNA was depleted by up to 85%, resulting in a lack of pupal viability.

Finally, in *polg* mutant nematodes, cell cycle arrest was evidenced at L2 and L3 larval stages. In these worms, mtDNA content was consistently decreased compared to controls, though the mutations used are entire exon removals, in contrast to clinically reported *POLG* variants.

### Risk of bias assessment

**Figure 2.**
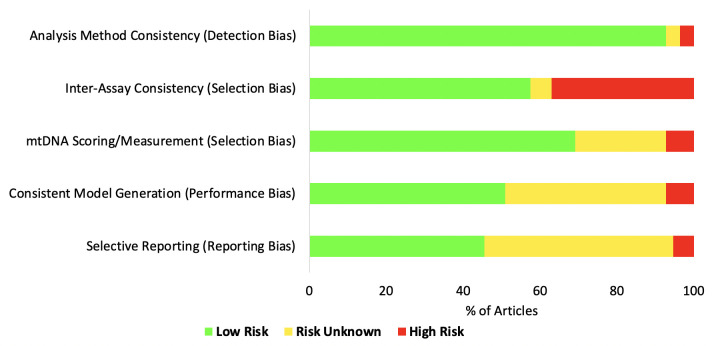
Risk of Bias analysis summary. ‘Risk of Bias’ for all articles included was assessed via an adapted Cochrane Risk of Bias tool ^8^. Each article was individually appraised (Supplementary Material, Table 6B–C [ *Extended data* ^5^]), with a judgement of the level of risk within an article and across each domain deemed as ‘low’, ‘unclear’ or ‘high’, according to a majority response to meeting pre-specified criteria (Supplementary Material, Table 6A [ *Extended data* ^5^]). The x-axis represents the total proportion of included articles in each bias category, while the y-axis represents each bias domain.

The adapted Cochrane Risk of Bias Tool assessment ( Table 2; Figure 2) demonstrated variability across all articles. Notably, model selection consistency within individual articles possessed a high risk of bias (selection bias domain) in articles using multiple assays ( Figure 2; Supplementary Material, Table 6C [ *Extended data* ^5^]). Other risk of bias domains were mainly reported as unclear ( Figure 2; Supplementary Material, Table 6C [ *Extended data* ^5^]), largely due to a lack of methodological reporting.

Regarding each model system, the only model at a high risk of bias was *C. elegans*, likely a consequence of its inconsistent use across a small number of studies (N = 2). All other models were appraised as low or unclear risk for all domains, with murine models the only model system deemed to be low risk for all 5 domains ( Table 2, Supplementary Material, Table 6D [ *Extended data* ^5^]).

## Discussion

This review provides a comprehensive summary of the evidence for the effectiveness of cell and animal models to recapitulate molecular, genetic, and/or phenotypic features of *POLG*-related disease. We found that no models are optimally established to recapitulate the full spectrum of *POLG*-related disease. While each of the models reviewed possess advantages and disadvantages, we are unable to definitively advocate for the use of any single model. In the absence of further studies, a multi-model approach may be necessary for meaningful preclinical studies of *POLG*-related disease. Furthermore, we have noted that the design and utilisation of *POLG* models appears to follow general research trends with, for example, iPSC-derived models becoming popular in recent years.

The risk of bias appraisal demonstrated significant concerns across the majority of model systems and their use in *POLG*-related research, predominantly due to poor reporting quality and inconsistent methodologies. Improving the methodological quality of articles to reduce repetition represents an important aspect in *POLG*-related research which should be considered by future investigators.

*S. cerevisiae* models provide a potential for high-throughput variant screening ^16^, due to the simplicity by which dysfunction in mtDNA replication can be analysed. Although largely a well-regarded model, *S. cerevisiae* is limited in its relevance to *POLG*-related disease, through its inability to assess tissue-specific phenotypes.

Murine models have allowed research into tissue-specific *POLG*-related disease through targeted transgenic genes. However, these models have limitations e.g. the Y955C pol γ murine model accurately recapitulates mtDNA depletion, but the phenotype observed in this mouse (cardiomyopathy and fibrosis) does not mirror human *POLG*-related disease ^33^.

While articles utilising nematode models were limited due to the removal of whole *POLG* exons (unlike variants identified in human disease), studies demonstrated that nematode models are potentially capable of recapitulating mtDNA depletion, such as that seen in clinical cases of *POLG*-related disease ^19, 27^. However, evidence of this is hampered by the small number of publications that utilised this model within the literature. Notably, the demonstration that CLO can rescue mtDNA depletion is a significant milestone amongst all of the reviewed articles as it is one of few potential treatments shown to exhibit some rescue of depleted mtDNA levels. In order to assess the ability of nematodes to more accurately model *POLG*-related disease, future research should utilise clinically relevant single nucleotide variants and assess the response to CLO.

Similar to nematodes, the sole zebrafish model employed was also limited by removal of entire coding sequences; a quite different situation to the single nucleotide variants commonly identified in patients with *POLG*-related disease ^31^. As the study did not involve the use of zebrafish with variants homologous to those seen in *POLG*-related disease, its clinical relevance is limited, and similarly to nematodes, requires further investigation using zebrafish engineered with single amino acid missense variants. This problem is further compounded by the inability of heterozygous variants to induce mtDNA depletion, as seen in human disease-causing *POLG* variants. Although Facchinello *et al.* ^30^ investigated the effects of CLO in rescuing mtDNA depletion in mutant *POLG* models, the effects observed, did not correlate with those of CLO in nematodes ^19^. This may be explained by the nature of the removal of whole exons.

Human cellular models were derived from reprogramming patient fibroblasts into iPSCs using retroviruses, before differentiation into hepatocyte-like cells, neural stem cells, neurons, and astrocytes. Although the increasing use iPSC models may reflect recent trends in research, these studies may provide a basis for development of organoid models of *POLG*-related disease. Organoid models are large 3D culture systems, derived from stem cells that act as ‘proto-organs’ in modelling, generated through specified culture induction ^34^. Although the use of iPSC-generated hepatocytes and neurons are now well established, the generation of hepatic or neural organoid models will add exciting, novel capabilities to the field’s quest for effective therapeutics ^35^.

Overall, phenotypic recapitulation of human *POLG*-related disease in the evaluated models was extremely limited. Although iPSC-based studies allowed differentiation into neurons and hepatocytes, no single model emerged as superior. From a molecular perspective, these models did typically exhibit mtDNA depletion, reflective of paediatric onset *POLG*-related disease ^3^. Of all articles reviewed: no studies were able to demonstrate therapeutic efficacy; none identified the previously reported sex bias ^36^; and many failed to provide useful mechanistic insights regarding *POLG*-related mitochondrial disease. These findings may in part be explained by the paucity of high-quality evidence provided in these articles.

## Conclusions

The combined benefits of the models identified in this review may support the future development of an algorithm for use in preclinical research into *POLG*-related disease. Although each model system possessed inherent limitations, the studies reported here set a foundation for future research to improve recapitulation of disease phenotypic expression and therefore increase the translational potential of promising experimental interventions. Few models interrogated the use of treatments, with only a small number of studies attempting to elucidate disease mechanisms and how they can modulate mitochondrial function.

## Data Availability

Figshare: Informing drug discovery: a systematic review of POLG-related disease models, https://doi.org/10.25405/data.ncl.21588042 ^5^ This project contains the following underlying data: Supplementary Table 5. Data extracted from all included articles. Supplementary Table 6A. Assessment of the reporting quality of each article. Supplementary Table 6B. The risk of bias of data for each model system type. Figshare: Informing drug discovery: a systematic review of POLG-related disease models, https://doi.org/10.25405/data.ncl.21588042 ^5^ This project contains the following extended data: Supplementary Figure 1: PRISMA Flow Diagram Supplementary Table 2. Search strategy. Supplementary Table 3. Detailed article inclusion/exclusion criteria. Supplementary Table 4. Excluded full text articles. Figshare: PRISMA checklist for “Model systems informing mechanisms and drug discovery: a systematic review of POLG-related disease models”, https://doi.org/10.25405/data.ncl.21588042 ^5^ Data are available under the terms of the Creative Commons Attribution 4.0 International license (CC-BY 4.0).
