# Supplementary material for: Model systems informing mechanisms and drug discovery: a review of POLG-related disease models
Source: Wellcome Open Res. 2025 Apr 4;8:33. Originally published 2023 Jan 20. [Version 2] doi: 10.12688/wellcomeopenres.18637.2 (PMC12586980; doi:10.12688/wellcomeopenres.18637.2)
Supplement: Peer review history

# Reviewer report: Reviewer response for version 2

**DOI:** 10.21956/wellcomeopenres.26516.r126718

**Published:** 2025-11-05

**Authors:** Wolfram S Kunz

**Affiliations:** 1 University of Bonn, Bonn, Germany

This review investigates reports of various models of POLG related disease regarding suitability for disease modeling and drug discovery. Although the paper is well written and in general sound there are the following problems to be considered.

 1. Quite recently very important papers about mice models [Reference 1] and therapeutic approaches [Reference 2] appeared which for obvious reasons have not been included. The authors should therefore consider to alter their very early search deadline (May 31st 2022). 

 2. Obviously, from a mechanistic molecular perspective, several points discussed in the paper seem obvious even without the performed sophisticated investigation. That relates especially to phenotype issues which are even in humans complicated (patients harboring the same mutations can have distinct phenotypes). Some model organisms simply do not have an accessory subunit of POLG, which has been found to be very relevant for phenotype severity [Reference 1].

 3. The authors focused on decreased mtDNA copy number as main molecular readout. In human disease multiple mtDNA deletions at normal mtDNA copy numbers also play an important role. This disease condition cannot be recapitulated in animal models so far. That problem should be at least mentioned and discussed.

Are the rationale for, and objectives of, the Systematic Review clearly stated?

Yes

Is the statistical analysis and its interpretation appropriate?

Yes

If this is a Living Systematic Review, is the ‘living’ method appropriate and is the search schedule clearly defined and justified? (‘Living Systematic Review’ or a variation of this term should be included in the title.)

Not applicable

Are sufficient details of the methods and analysis provided to allow replication by others?

Yes

Are the conclusions drawn adequately supported by the results presented in the review?

Partly

Reviewer Expertise:

Mechanisms of mtDNA turnover

I confirm that I have read this submission and believe that I have an appropriate level of expertise to confirm that it is of an acceptable scientific standard, however I have significant reservations, as outlined above.

## References

[1] : Modelling POLG mutations in mice unravels a critical role of POLγΒ in regulating phenotypic severity. *Nature Communications* .2025;16(1) : 10.1038/s41467-025-60059-y 10.1038/s41467-025-60059-y PMC1209891640404629

[2] : Small molecules restore mutant mitochondrial DNA polymerase activity. *Nature* .2025;642(8067) : 10.1038/s41586-025-08856-9 501-507 10.1038/s41586-025-08856-9 40205042 PMC12158775

# Reviewer report: Reviewer response for version 2

**DOI:** 10.21956/wellcomeopenres.26516.r126710

**Published:** 2025-11-04

**Authors:** Ann Saada

**Affiliations:** 1 Hebrew University, Jerusalem, Israel

This review summarizes the current status of POLG research models and their usefulness in recapitulating human POLG- related diseases. It is an important contribution to ongoing efforts to elucidate the pathomechanisms underlying the broad phenotypic spectrum of POLG disorders. The authors have satisfactorily addressed the constructive comments of the two reviewers. I therefore have only a few additional remarks:

 1. The initial search yielded approximately 30,000 articles, from which only 55 were included in the final synthesis. It would be helpful to specify the keywords used in the initial search. Additionally, it would be interesting to know whether applying narrower search terms would still yield the same 55 included articles.

 2. In the discussion, the conclusions are framed in relation to POLG diseases in general. However, there may be specific correlations between certain models and defined clinical phenotypes, such as Alpers–Huttenlocher syndrome (AHS), mitochondrial recessive ataxia syndrome (MCHS), myoclonic epilepsy myopathy sensory ataxia (MEMSA), ataxia neuropathy spectrum (ANS), or autosomal recessive/dominant progressive external ophthalmoplegia (ar/ad PEO). Such correlations could be valuable for selecting appropriate models for variant validation and drug evaluation.

 3. Consider discussing the role of POLG2 (encoding the accessory subunit POLG-β) in modulating phenotypic severity, particularly in light of the recent findings by Corrà et al. (Reference 1).

Are the rationale for, and objectives of, the Systematic Review clearly stated?

Yes

Is the statistical analysis and its interpretation appropriate?

Yes

If this is a Living Systematic Review, is the ‘living’ method appropriate and is the search schedule clearly defined and justified? (‘Living Systematic Review’ or a variation of this term should be included in the title.)

Not applicable

Are sufficient details of the methods and analysis provided to allow replication by others?

Yes

Are the conclusions drawn adequately supported by the results presented in the review?

Partly

Reviewer Expertise:

Mitochondrial Respiratory Chain, Mitochondrial diseases, Inborn Errors of Metabolism

I confirm that I have read this submission and believe that I have an appropriate level of expertise to confirm that it is of an acceptable scientific standard.

## References

[1] : Modelling POLG mutations in mice unravels a critical role of POLγΒ in regulating phenotypic severity. *Nature Communications* .2025;16(1) : 10.1038/s41467-025-60059-y 10.1038/s41467-025-60059-y PMC1209891640404629

# Reviewer report: Reviewer response for version 2

**DOI:** 10.21956/wellcomeopenres.26516.r122406

**Published:** 2025-05-23

**Authors:** Ana Cristina Andreazza

**Affiliations:** 1 Department of Pharmacology and Toxicology, Faculty of Medicine, University of Toronto, Toronto, ON, Canada

Thank you for providing me with the opportunity to review your manuscript entitled "Model systems informing mechanisms and drug discovery: a review of POLG-related disease models."

 I have responded positively to all the relevant questions (rationale and objectives, methodological details, statistical analysis, and conclusions). Your systematic review clearly articulates the rationale and objectives, provides sufficient methodological details for reproducibility, applies appropriate statistical interpretation, and draws conclusions supported adequately by your presented results.

 I have two small suggestions:

1. **Phenotypic Recapitulation**: Although your review comprehensively covers molecular and genetic recapitulation, phenotypic recapitulation appears to be limited across models. DO you think you could perhaps clarify the challenges and potential strategies or recommendations to improve phenotypic recapitulation in future studies.
2. **Therapeutic Evaluations**: You briefly discuss therapeutic explorations but highlight their limited scope and mixed results. DO you think you could provide  more detailed recommendations or strategies on how future research could approach therapeutic interventions in POLG-related models more effectively? 

Are the rationale for, and objectives of, the Systematic Review clearly stated?

Yes

Is the statistical analysis and its interpretation appropriate?

Yes

If this is a Living Systematic Review, is the ‘living’ method appropriate and is the search schedule clearly defined and justified? (‘Living Systematic Review’ or a variation of this term should be included in the title.)

Not applicable

Are sufficient details of the methods and analysis provided to allow replication by others?

Yes

Are the conclusions drawn adequately supported by the results presented in the review?

Yes

Reviewer Expertise:

Mitochondrial research

I confirm that I have read this submission and believe that I have an appropriate level of expertise to confirm that it is of an acceptable scientific standard.

# Reviewer report: Reviewer response for version 1

**DOI:** 10.21956/wellcomeopenres.20666.r57979

**Published:** 2023-06-22

**Authors:** Johannes K Ehinger, Emil Westerlund

**Affiliations:** 1 Lund University, Lund, Sweden

The authors set out to systematically describe the available pre-clinical models of POLG-related disease, a rare primary mitochondrial disease that results in mitochondrial DNA (mtDNA) deletions and depletion due to pathogenic variants in the catalytic subunit of the human polymerase gamma. They take a systematic approach to identifying relevant literature and frame their work as a systematic review.

 The protocol is registered in Prospero, as should be for a systematic review. The reason for a protocol of a systematic review to be published prior to the execution of the study is to avoid bias in terms of selective reporting. Hence a published protocol should be as detailed as possible, and any deviation from the protocol should be explained in the manuscript. In this case, there are substantial deviations from the protocol in the manuscript that are not explained, which must be amended before any further evaluation of the manuscript can be made.

 The protocol is written in a very general way, and I do recognize that covering the whole thematic field included in the protocol is difficult in one manuscript, but this is a methodological flaw and as such needs to be addressed as outlined below. The deviations are of such magnitude that they can’t all be detailed, and they will hence here be described in general terms, with some specific examples.

 The review questions outlined in the protocol are as below:

1. What are the clinical features/syndromes (typical and atypical phenotypes) known to be associated with specific POLG pathogenic variants and how do they affect disease progression?
2. What are the genetic or environmental factors that influence the phenotypic expression of POLG disease (e.g. haplotype, hormones, nutritional status, exercise, viral illness)?
3. What are the molecular genetic mechanisms underpinning POLG-related disorders?
4. What current treatments exist for the managements of POLG-related disorders and what prospective treatments are in development?

In the manuscript, the described aim of the study is:

- To close this knowledge gap, we sought to systematically investigate the effectiveness of cell and animal models to recapitulate features of POLG-related disease, from a molecular, genetic, and phenotypic perspective

While the above is an entirely valid research question, it is not one of the review questions outlined in the protocol.

 Is it so that this protocol is intended to result in several separate reviews? If that is the case that should ideally have been defined in the protocol, and most definitely needs to be addressed in the manuscript. The protocol states two inclusion criteria, one relating to reports from patients and one relating to animal and cell models. It is rather clear that this current manuscript focuses on just the latter of these two. This deviation from the protocol needs to be addressed in the manuscript. In the manuscript, it states that “Articles relating to the POLG D257A ‘mutator’ mouse model were exclude”. This exclusion is not defined in the protocol and hence needs to be reported as a deviation from the protocol. Further, when comparing the full inclusion and exclusion criteria in the protocol and Table S3, these are not the same.

 The general feeling here is that the authors are eager to frame this a systematic review, while the subject in itself is ill suited for this methodology. I would suggest to simply label this as a scoping review instead, as this is more in line with what has actually been done. Similar to a systematic review, a scoping review have a systematic approach, with defined search strategies, but the purpose of a scoping review is to map the literature on a topic area, while a systematic review analyses the best available research on a specific question. By attempting to label this as a systematic review, the authors are essentially “asking for” a lot of criticism that in essence is not about the validity of the review, but rather just the unfortunate choice of review type at the project inception. My suggestion would be to call this a scoping review instead, and simply describe the process, that initially this was a project with X, Y and Z research questions, and that you took a systematic approach, but due to the body of literature found, instead you focused on question A (and B and C in separate reviews) and that this caused substantial protocol deviation. The alternative is to throughout the manuscript address all the protocol deviations which will make the manuscript rather messy and won’t benefit the reader.

 To just provide a few more examples relating to the above, if this is actually a systematic review looking at “the effectiveness of cell and animal models to recapitulate features of POLG-related disease, from a molecular, genetic, and phenotypic perspective”, then you also would have to provide a definition about how you measure this effectiveness, a that must be done a priori in the protocol and otherwise reported as a protocol deviation. It is difficult to see that the main outcomes outlined in the protocol are those that actually are used in the manuscript. In table 2, the claim “Models were deemed as Yes/Unclear/No/Not applicable (N/A) in relation to their effectiveness to meet pre-defined criteria.” These pre-defined criteria can’t be found in the protocol.

 In the section regarding statistical analysis one can read:

- “The methodological quality of the included studies was limited, with variability of extracted data precluding a meta-analysis. Compatible results across model systems were pooled for interpretation.”

This is not detailed enough to allow for replication and must be better described.

**Minor comments:**

- Reference 2 is not an original epidemiological study, please reference original research in support of the claim that POLG is the most common cause of MD.
- Percentages are redundant when the total number is less then 100 as e.g., in “results overview”, I suggest just reporting natural frequencies.

This peer review is not exhaustive, due to the need to rework the study in relation to its protocol, and needs to be reviewed again in its next version.

Are the rationale for, and objectives of, the Systematic Review clearly stated?

No

Is the statistical analysis and its interpretation appropriate?

Not applicable

Are sufficient details of the methods and analysis provided to allow replication by others?

No

Are the conclusions drawn adequately supported by the results presented in the review?

No

Reviewer Expertise:

NA

We confirm that we have read this submission and believe that we have an appropriate level of expertise to state that we do not consider it to be of an acceptable scientific standard, for reasons outlined above.

# Response

**Published:** 2025-03-21

**Authors:** Renae Stefanetti

**Affiliations:** Newcastle University, UK

The following is a response to Prof. Johannes K Ehinger and Emil Westerlund, who have both kindly provided feedback and comments on our review of literature in the field of *POLG*-related disease research.  

**Comment 1:** *The protocol is registered in Prospero, as should be for a systematic review. The reason for a protocol of a systematic review to be published prior to the execution of the study is to avoid bias in terms of selective reporting. Hence a published protocol should be as detailed as possible, and any deviation from the protocol should be explained in the manuscript. In this case, there are substantial deviations from the protocol in the manuscript that are not explained, which must be amended before any further evaluation of the manuscript can be made.* *The protocol is written in a very general way, and I do recognize that covering the whole thematic field included in the protocol is difficult in one manuscript, but this is a methodological flaw and as such needs to be addressed as outlined below. The deviations are of such magnitude that they can’t all be detailed, and they will hence here be described in general terms, with some specific examples.* *“To close this knowledge gap, we sought to systematically investigate the effectiveness of cell and animal models to recapitulate features of POLG-related disease, from a molecular, genetic, and phenotypic perspective” While the above is an entirely valid research question, it is not one of the review questions outlined in the protocol.* *Is it so that this protocol is intended to result in several separate reviews? If that is the case that should ideally have been defined in the protocol, and most definitely needs to be addressed in the manuscript.* *The protocol states two inclusion criteria, one relating to reports from patients and one relating to animal and cell models. It is rather clear that this current manuscript focuses on just the latter of these two. This deviation from the protocol needs to be addressed in the manuscript. In the manuscript, it states that “Articles relating to the POLG D257A ‘mutator’ mouse model were exclude”. This exclusion is not defined in the protocol and hence needs to be reported as a deviation from the protocol. Further, when comparing the full inclusion and exclusion criteria in the protocol and Table S3, these are not the same.* *The general feeling here is that the authors are eager to frame this a systematic review, while the subject in itself is ill suited for this methodology. I would suggest to simply label this as a scoping review instead, as this is more in line with what has actually been done. Similar to a systematic review, a scoping review have a systematic approach, with defined search strategies, but the purpose of a scoping review is to map the literature on a topic area, while a systematic review analyses the best available research on a specific question. By attempting to label this as a systematic review, the authors are essentially “asking for” a lot of criticism that in essence is not about the validity of the review, but rather just the unfortunate choice of review type at the project inception.* *My suggestion would be to call this a scoping review instead, and simply describe the process, that initially this was a project with X, Y and Z research questions, and that you took a systematic approach, but due to* *the body of literature found, instead you focused on question A (and B and C in separate reviews) and that this caused substantial protocol deviation.* *The alternative is to throughout the manuscript address all the protocol deviations which will make the manuscript rather messy and won’t benefit the reader.* *To just provide a few more examples relating to the above, if this is actually a systematic review looking at “the effectiveness of cell and animal models to recapitulate features of POLG-related disease, from a molecular, genetic, and phenotypic perspective”, then you also would have to provide a definition about how you measure this effectiveness, a that must be done a priori in the protocol and otherwise reported as a protocol deviation. It is difficult to see that the main outcomes outlined in the protocol are those that actually are used in the manuscript. In table 2, the claim “Models were deemed as Yes/Unclear/No/Not applicable (N/A) in relation to their effectiveness to meet pre-defined criteria.” These pre-defined criteria can’t be found in the protocol.*

**Response 1:** We sincerely appreciate the reviewer’s thorough evaluation and constructive feedback. We acknowledge that the original PROSPERO registration encompassed a broad scope, intended to inform multiple reviews. Given the complexity of systematically assessing *POLG*-related disease models, our approach in this manuscript naturally led to deviations from the initial protocol, particularly in focusing on research question 3. To address these concerns, we have reframed the manuscript to better reflect its methodological approach. Specifically, we have removed references to this work as a "systematic review" and instead clarified that we employed a "systematic approach wherever possible." This change is reflected in the revised title: *"Model systems informing mechanisms and drug discovery: a review of POLG-related disease models"* We recognise the importance of transparently reporting protocol deviations. Accordingly, we have updated the manuscript to explicitly outline these deviations and provide justification for the refined scope. Additionally, we have amended the manuscript throughout to align with the methodological approach taken in this review, ensuring that any exclusions (such as the *POLG *D257A mutator mouse model) and modifications to inclusion/exclusion criteria are fully addressed. We appreciate the reviewer's suggestion that a scoping review may be a more appropriate classification. While our approach retained key systematic elements, including a structured search strategy and predefined eligibility criteria, the refinement of our research focus aligns more closely with a scoping review methodology. We have therefore clarified this distinction in the manuscript to prevent misinterpretation of our approach. We hope these revisions adequately address the concerns raised and improve the clarity and transparency of our methodology. We appreciate the opportunity to strengthen our work based on the reviewer's insightful feedback. Deviations from Protocol During development of the review, several substantial deviations were made from the original PROSPERO-registered protocol. These deviations are listed here, along with their rationale: *Review question –* The registered PROSPERO protocol initially aimed to address four research questions. However, this review is the focus of a single question (Question 3: *What are the molecular genetic mechanisms underpinning POLG-related disorders*). To enhance clarity, this question was rephrased to specifically investigate the effectiveness of cell and animal models recapitulating molecular, genetic, and phenotypic features of *POLG*-related disease. *Type and method of review – *This original protocol outlines a systematic review. However, given the nature and scope of the available literature, it was appropriate to deviate from the approach. Instead, the current review encompassed a scoping review while employing  systematic elements wherever possible, including a systematic literature search and a risk of bias assessment. *Eligibility criteria –* Inclusion criteria: The registered protocol included human-based studies as an inclusion criterion. However, in accordance with the revised research question, only studies utilising animal and/or cellular models were included. Therefore, human-based studies were removed from the inclusion criteria. Exclusion criteria: Not included in the registered protocol, studies utilising the *polg*-D257A mutator mouse model were excluded. During the literature screening phase , thorough discussion among investigators determined that this model does not accurately model does not reflect clinical *POLG*-related disease. Therefore, studies utilising the *polg*-D257A mutator mouse model were excluded to prevent misrepresentation of the animal and cellular models of clinical *POLG*-related disease. The eligibility criteria applied in this review are  detailed in the Supplementary Material, Table 3 [Extended data ^5^]. *Data extraction and synthesis – * The registered protocol included data extraction of human-based studies. In line with the revised research question, data were extracted exclusively from  studies including cellular/animal models. Furthermore, the registered protocol did not detail how the effectiveness of different models would be assessed. To address this, molecular indications (e.g., mtDNA levels) and exhibited phenotypes (e.g., myopathy) were evaluated across included studies (see Supplementary Material, Table 3 [Extended data ^5^]). Any affected measures were reported for each included article (see Supplementary Material, Table 5 [ *Extended data* ^5^]) and subsequently aggregated across articles to establish a majority-based consensus on the extent to which these measures recapitulated features of *POLG*-related disease (Table 2).

**Comment 2:** *In the section regarding statistical analysis one can read: “The methodological quality of the included studies was limited, with variability of extracted data precluding a meta-analysis. Compatible results across model systems were pooled for interpretation.” This is not detailed enough to allow for replication and must be better described.*

**Response 2:**    We acknowledge the need for greater clarity in the statistical analysis description. We have revised the section to state: " *Compatible results across model systems, such as evidence of common phenotypes or quantifiable levels of mtDNA depletion, were pooled for interpretation."* This revision provides concrete examples of how pooling was conducted, facilitating replication.

**Comment 3:** *Minor comments:* *Reference 2 is not an original epidemiological study, please reference original research in support of the claim that POLG is the most common cause of MD.*

**Response 3:**    We appreciate this suggestion. Reference 2 has been updated to refer to Barca et al. (2020), "Mitochondrial diseases in North America: An analysis of the NAMDC Registry."

**Comment 4:** *Percentages are redundant when the total number is less then 100 as e.g., in “results overview”, I suggest just reporting natural frequencies.*

**Response 4:**    We understand the reviewer’s perspective and recognise the potential redundancy of percentages in small sample sizes. However, we have retained both natural frequencies and percentages to maintain clarity for all readers. This ensures accessibility for those who prefer percentage-based representation, as is common in published literature.

**Comment 5:** *This peer review is not exhaustive, due to the need to rework the study in relation to its protocol, and needs to be reviewed again in its next version.*

**Response 5:**    We thank the reviewer for their time and constructive feedback. We appreciate the opportunity to revise our manuscript and believe these revisions address all concerns while ensuring clarity and transparency in our methodology. Thank you again for your valuable insights.

# Reviewer report: Reviewer response for version 1

**DOI:** 10.21956/wellcomeopenres.20666.r54452

**Published:** 2023-02-06

**Authors:** Simon Johnson

**Affiliations:** 1 Applied Science, Translational Bioscience, Northumbria University, Newcastle, UK

This is a review of literature in the field of POLG related disease research.

 While the topic is of high interest and the review warranted, there are many issues that will need to be addressed prior to indexing:

1. The inclusion/exclusion for manuscripts assessed are not completely clear, and in some cases decisions not well justified. For example, exclusion of POLG mutator mice, which is used in biogerontology as a 'rapid aging' models, is not warranted. This model shows symptoms of POLG disease and is a direct model of POLG deficiency in mammals.
2. The overview of 'risks of biases' is not well constructed. It is not clear how risks are judged, how entire groups of articles can be ranked together, or what value this provides when all scores are 'low' or 'unknown.' My suggestion is a complete removal - I'm not sure how it can be revised in a way that provides a useful addition to the review.
3. This reviewer strongly feels a more detailed discussion of how different models actually represent disease is necessary. What endpoints are used in what models, and what symptoms/disease sequelae can be studied in which model systems. iPSC's are hailed as 'possibly the most advanced models...a gold-standard' but this is not a view I think most POLG researchers would agree with. Isolated cells, even if genetically identical to patients, cannot model complex multi-system disease. Even organoids cannot model complex multi-system disease, and while currently popular, they are an as yet uproven technology in this field (no iPSC organoid work has not led to significant advances in POLG research that I'm aware of).
4. An overview of phenotypes in the animal models and how they relate to humans is necessary.
5. The key points in the title are not really directly addressed in the manuscript - mechanisms and drug discovery. There should be sections, at least in the discussion, specifically addressing these two points as they are the focus of the review title.
6. The review overall lacks a synthesis in the conclusions. Do the authors have any suggestions for improvement of future studies? Have any models provided a 'best' approximation of human disease? Where do intervention studies lie? What endpoints are rigorous, and which should be avoided? This reviewer would like to see a discussion of practical/technical aspects of POLG work as well - what are the best assays for mtDNA depletion, etc. So far, the review provides a partial summary, but lacks a real synthesis of data.

There are also a number of errors, such as the use of 'Nematoda' whenever *C. elegans* data are referenced. Nematoda is the Phylum, and Nematoda as a phylum are not the model system. The accepted way to discuss *C. elegans *data is to use the species name or to call them nematodes as a generic term, not to use the phylum name.

Additional comments are as follows:

 Less than 1% of all articles were included. I feel that more rigorous/specific search terms would have been a better approach than identifying ~30,000 articles and manually sorting out less than 1% for inclusion in this review – only 55 included in the qualitative synthesis. This does not lead to confidence in the claim that this is a ‘systematic’ review. It would be impossible for a reviewer or reader to sort through the 30,000 and determine whether the authors were in fact systematic about their approach. I would much prefer reporting of a more specific set of search terms that results in a higher % of articles included in the review.

 Abstract, last sentence: “Here, we sought to perform a systematic review of the existing models used in mitochondrial research and their effectiveness in recapitulating POLG-related disease.” ‘models used in mitochondrial research’ is too broad – the authors only review models of PolG related disease. I suggest revising this to “Here, we sought to perform a systematic review of the existing models used in POLG-related disease research and their effectiveness in recapitulating human pathologies.”

 I suggest revising the first sentence of the last paragraph of the abstract “The available evidence supporting the ability of models for POLG-related disease to recapitulate molecular mechanisms and phenotype is limited, inconsistent and of poor methodologic quality.” Perhaps say ‘much is of poor quality’. I agree with the overall sentiment the authors are conveying, but it is probably best to not infer that *all* research in the area is of poor quality. They authors also need to back up this statement a bit more clearly. It may be true, but nothing in the text explicitly discusses quality issues in the literature or how the authors arrived at this conclusion. 

 Minor grammatical errors throughout – most often extra commas or improper use of semicolons. Please carefully revise.

 “In order to understand the molecular mechanisms underlying *POLG*-related disease and inform future therapeutic targets, researchers have created disease models, despite a paucity of supporting evidence.” – not clear what the last 6 word stretch of this sentence is trying to convey. These models were developed in order to address a lack of evidence and contribute to our basic understanding of POLG-related diseases, no?

 The so-called ‘progeroid’ POLG mutator mouse is excluded, but the decision to exclude these studies is not rational. Some phenotypes certainly overlap, and they probably provide a much closer model for POLG disease in humans than some of the models that are included. Is the myopathy seen in mutator mice not relevant? I’m really not convinced that this model is not a model for mitochondrial disease. To me, it is its value as an accelerated aging model that is questionable. POLG defects in humans cause mitochondrial disease, not progeria. POLG defects related to the mutator mice cause cardiomyopathy, which is specifically discussed by the authors as a good sign of recapitulation of human POLG disease.

 “While translating would remove bias, articles that are robust and of high impact are likely to be published in an English language journal” is somewhat offensive to non-English journals/researchers. I recommend re-thinking the language here.

 In Table 1, please change ‘women/men’ to ‘female/male’ – I’ve never heard of mice being called ‘women/men’. Also of concern – nematodes are listed as 50/50 ‘women/men’ – as I’m sure the authors are aware, *C. elegans* are either male (rarely) or hermaphrodite (commonly). The sexes cannot and should not be summed as they have been. A male human and male *C elegans* are not remotely equivalent.

 As ‘petite colonies’ only applies to yeast, I’m not sure separating it into three levels is warranted in this synthesis. All endpoints that appear in one organism could probably be collapsed into an ‘other’ or ‘model specific’ category and detailed individually.

 There are serious issues with the ‘bias’ assessment portion of this manuscript. I don’t feel the ‘risk of bias’ application is clearly detailed or (without very clear details regarding how the Cochrane Risk of Bias tool was ‘adapted’) even reasonable for the types of articles reported. It is also completely unclear how a combined risk of bias can be included for all manuscripts on a given model – for example, there is one risk score for interassay variability for all human data (marked as ‘low’). This is not reasonable. The review would be better off without any ‘risk of bias’ assessment than with one that seems so highly at risk for bias itself. The authors also score everything as low or unclear. It is unclear what value the bias assessment has if everything scores the same way. How are mice and iPSC’s determined to be ‘low’ risk of selective reporting bias? I don’t see how that conclusion can be drawn. How is everything else unknown?

 Why is *C. elegans* described in the text as ‘Nematoda’? The entire phylum of Nematoda is not used as a model.

 The ‘Molecular Recapitulation’ section should simply focus on 1) does the genetic manipulation / model accurately represent the genetic lesions that occur in humans and 2) do the molecular consequences of the manipulation/model accurately represent those occurring in humans? The authors include discussion of lower mtDNA in aging worms in this section, while they’ve eliminated the POLG mutator mouse studies from this review because they ‘model aging’. These are incompatible decisions.

 Some remarks should be provided regarding quality of assays – for example, standard qPCR is no longer a good option if quantitative data regarding mtDNA copy numbers is desired.

 “Generally causing cell cycle arrest at the L2 and L3 larval stages due to insufficient mtDNA levels.” This is a model, there is no evidence that convincingly demonstrates that it is a lack of mtDNA *per se* that leads to arrest.

 What is the proposed mechanism for Clofilium tosylate? If a mechanism is not discussed, the drug should not even be mentioned.

 Why would iPSC’s be considered a ‘gold standard’ over *in vivo* mouse models? I don’t think this is a view that anyone studying disease pathogenesis would agree with. Molecular features and cell autonomous features may be most directly modeled in iPSC’s, but features of disease pathology in tissue and in multi-system disease cannot even be approached. An ability to alter mtDNA levels in iPSC’s may not extend to in vivo relevance.

 The ”Sirt1 (mitophagy) pathway” is not a discrete/clear thing. Details need to be provided.

Are the rationale for, and objectives of, the Systematic Review clearly stated?

Partly

Is the statistical analysis and its interpretation appropriate?

Not applicable

Are sufficient details of the methods and analysis provided to allow replication by others?

No

Are the conclusions drawn adequately supported by the results presented in the review?

Partly

Reviewer Expertise:

Mitochondrial disease

I confirm that I have read this submission and believe that I have an appropriate level of expertise to state that I do not consider it to be of an acceptable scientific standard, for reasons outlined above.

# Response

**Published:** 2025-03-21

**Authors:** Renae Stefanetti

**Affiliations:** Newcastle University, UK

We sincerely appreciate Dr Johnson’s thorough review and constructive feedback on our review of *POLG*-related disease models. Below, we address each comment with clarity and appropriate revisions where necessary.  

**Comment 1:** *The inclusion/exclusion for manuscripts assessed are not completely clear, and in some cases decisions not well justified. For example, exclusion of POLG mutator mice, which is used in biogerontology as a 'rapid aging' models, is not warranted. This model shows symptoms of POLG disease and is a direct model of POLG deficiency in mammals.* *The overview of 'risks of biases' is not well constructed. It is not clear how risks are judged, how entire groups of articles can be ranked together, or what value this provides when all scores are 'low' or 'unknown.' My suggestion is a complete removal - I'm not sure how it can be revised in a way that provides a useful addition to the review.*

**Response 1:** We thank the reviewer for their comment. These criteria were pre-specified (PROSPERO registered), formulated and justified within **Supplementary Material, Table 3 **To clarify the rationale for exclusion of articles solely related to the D257A mutator mouse, as well as the overall deviations from the registered PROSPERO protocol, a section noe detailed in the **Methods** section.  We maintain that the risk of bias assessment is a fundamental aspect of this thorough review. Our methodology follows Cochrane Collaboration recommendations. Many domains are ‘unclear’ due to poor reporting in preclinical studies, a recognised issue in the field. We have provided further details on our adaptation of the Cochrane Risk of Bias tool in **Supplementary Material, Table 6A **to enhance transparency.

**Comment 2:** *This reviewer strongly feels a more detailed discussion of how different models represent disease is necessary. What endpoints are used in what models, and what symptoms/disease sequelae can be studied in which model systems. iPSC's are hailed as 'possibly the most advanced models...a gold-standard' but this is not a view I think most POLG researchers would agree with. Isolated cells, even if genetically identical to patients, cannot model complex multi-system disease. Even organoids cannot model complex multi-system disease, and while currently popular, they are an as yet unproven technology in this field (no iPSC organoid work has not led to significant advances in POLG research that I'm aware of).*

**Response 2:** We thank the reviewer for their comment. As with systematic approaches, we have been exceptionally careful to be nuanced in our conclusions rather than narrative in judgements. To improve clarity, we have made a minor revision to the **Discussion **section accordingly: *“* *Overall, phenotypic recapitulation of human POLG-related disease in the evaluated models was extremely limited. Although iPSC-based studies allowed differentiation into neurons and hepatocytes, no single model emerged as superior. From a molecular perspective, these models did typically exhibit mtDNA depletion, reflective of paediatric onset POLG-related disease ^3^. Of all articles reviewed: no studies were able to demonstrate therapeutic efficacy; none identified the previously reported sex bias ^36^; and many failed to provide useful mechanistic insights regarding POLG-related mitochondrial disease. These findings may in part be explained by the paucity of high-quality evidence provided in these articles. **”*** Additionally, references to iPSCs as a ‘gold-standard’ in the **Discussion** and **Table 2 (footnote b legend)** have been removed to avoid any unintended implications. We have also amended **Table 2 (footnote b legend)**: *“All investigators agreed that it was important to consider iPSC models separately, based on their emergence as a distinct disease-specific cellular model in bioresearch ^12^.”*

**Comment 3:** *An overview of phenotypes in the animal models and how they relate to humans is necessary. The key points in the title are not really directly addressed in the manuscript - mechanisms and drug discovery. There should be sections, at least in the discussion, specifically addressing these two points as they are the focus of the review title.*

**Response 3: ** We thank the reviewer for this helpful suggestion. In order to address the relevance of the phenotypes, **paragraph 4** of the **Discussion** now reads “ *However, these models have limitations e.g. the Y955C polg murine model accurately recapitulates mtDNA depletion, but the phenotype observed in this mouse (cardiomyopathy and fibrosis) does not mirror human POLG-related disease ^35^.”* Additionally, **paragraph 5** has been changed to *“studies demonstrated that nematode models are potentially capable of recapitulating mtDNA depletion, such as that seen in clinical cases of POLG-related disease ^23,28^.”*

**Comment 4:** *The review overall lacks a synthesis in the conclusions. Do the authors have any suggestions for improvement of future studies? Have any models provided a 'best' approximation of human disease? Where do intervention studies lie? What endpoints are rigorous, and which should be avoided? This reviewer would like to see a discussion of practical/technical aspects of POLG work as well - what are the best assays for mtDNA depletion, etc. So far, the review provides a partial summary, but lacks a real synthesis of data*.

**Response 4:** We thank the reviewer for their suggestion. Notwithstanding the importance of the summary points raised, a narrative discussion is not within the remit of our scoping review, which employed systematic approaches wherever possible. Additionally, narrative reviews have already been published on mtDNA copy number techniques (e.g. PMID: 32004343).

**Comment 5:** *There are also a number of errors, such as the use of 'Nematoda' whenever C. elegans data are referenced. Nematoda is the Phylum, and Nematoda as a phylum are not the model system. The accepted way to discuss C. elegans data is to use the species name or to call them nematodes as a generic term, not to use the phylum name.*

**Response 5**: We thank the reviewer for this comment and have meticulously checked throughout the manuscript and revised accordingly.

**Comment 6:** *Less than 1% of all articles were included. I feel that more rigorous/specific search terms would have been a better approach than identifying ~30,000 articles and manually sorting out less than 1% for inclusion in this review – only 55 included in the qualitative synthesis. This does not lead to confidence in the claim that this is a ‘systematic’ review. It would be impossible for a reviewer or reader to sort through the 30,000 and determine whether the authors were in fact systematic about their approach. I would much prefer reporting of a more specific set of search terms that results in a higher % of articles included in the review.* *Abstract, last sentence: “Here, we sought to perform a systematic review of the existing models used in mitochondrial research and their effectiveness in recapitulating POLG-related disease.” ‘models used in mitochondrial research’ is too broad – the authors only review models of PolG related disease. I suggest revising this to “Here, we sought to perform a systematic review of the existing models used in POLG-related disease research and their effectiveness in recapitulating human pathologies.”* *I suggest revising the first sentence of the last paragraph of the abstract “The available evidence supporting the ability of models for POLG-related disease to recapitulate molecular mechanisms and phenotype is limited, inconsistent and of poor methodologic quality.” Perhaps say ‘much is of poor quality’. I agree with the overall sentiment the authors are conveying, but it is probably best to not infer that all research in the area is of poor quality. They authors also need to back up this statement a bit more clearly. It may be true, but nothing in the text explicitly discusses quality issues in the literature or how the authors arrived at this conclusion*. 

**Response 6**: As a scoping review, our study does not provide broad narrative conclusions but instead synthesises evidence based on predefined criteria and methodological rigour, ensuring transparency, reproducibility, and minimisation of bias, making our findings robust and reliable within the scope of the included evidence. We strongly disagree with the notion that the search strategy was not rigorous. The search strategy (overseen by an experienced medical sciences librarian) was designed to achieve maximum sensitivity (recall), therefore produced a very high number of results. It was never the aim to ‘sort through the 30,000 and determine whether the authors were in fact systematic about their approach’; our investigational team were prepared to screen this number of records at the title/abstract level for relevant studies for comprehensiveness, only those studies which made to the ‘inclusion’ stage were then quality assessed. This approach follows Cochrane Collaboration recommendations (Cochrane Handbook for Systematic Reviews of Interventions. Chapter 4). We acknowledge the reviewer’s point and have ensured that our findings explicitly highlight gaps in the field, referring readers to narrative reviews for additional methodological guidance. We have revised the **Abstract** to highlight the systematic approach to the review in the **first paragraph**: *“Here, we sought to employ systematic approaches, wherever possible, to investigate the cellular and animal models used in POLG-related research and assess how well they help us understand disease mechanisms in patients.” *We have also amended the **last paragraph** accordingly: *“* *While some evidence is available to support the ability of POLG-related disease models to recapitulate molecular mechanisms and phenotypes, much is of limited quality, with inconsistencies evident across the literature.”*

**Comment 7:** *Minor grammatical errors throughout – most often extra commas or improper use of semicolons. Please carefully revise.*

**Response 7**: The minor grammatical errors have been carefully revised and corrected throughout.

**Comment 8:** “ *In order to understand the molecular mechanisms underlying POLG-related disease and inform future therapeutic targets, researchers have created disease models, despite a paucity of supporting evidence.” – not clear what the last 6 word stretch of this sentence is trying to convey. These models were developed in order to address a lack of evidence and contribute to our basic understanding of POLG-related diseases, no?*

**Response 8:** We thank the reviewer for drawing this to our attention and have amended the sentence as follows: *“In order to understand the molecular mechanisms underlying POLG-related disease and inform future therapeutic targets, researchers have attempted to create models that recapitulate human disease, but evidence that these models accurately reflect human POLG-related disease is lacking.”*

**Comment 9:** *The so-called ‘progeroid’ POLG mutator mouse is excluded, but the decision to exclude these studies is not rational. Some phenotypes certainly overlap, and they probably provide a much closer model for POLG disease in humans than some of the models that are included. Is the myopathy seen in mutator mice not relevant? I’m really not convinced that this model is not a model for mitochondrial disease. To me, it is its value as an accelerated aging model that is questionable. POLG defects in humans cause mitochondrial disease, not progeria. POLG defects related to the mutator mice cause cardiomyopathy, which is specifically discussed by the authors as a good sign of recapitulation of human POLG disease.*

**Response 9:** We would respectfully disagree with the reviewer’s assertation regarding the *polg* mutator mouse and have outlined our reasons for excluding this model in **Response 1**.

**Comment 10:** “ *While translating would remove bias, articles that are robust and of high impact are likely to be published in an English language journal” is somewhat offensive to non-English journals/researchers. I recommend re-thinking the language here. In Table 1, please change ‘women/men’ to ‘female/male’ – I’ve never heard of mice being called ‘women/men’. Also of concern – nematodes are listed as 50/50 ‘women/men’ – as I’m sure the authors are aware, C. elegans are either male (rarely) or hermaphrodite (commonly). The sexes cannot and should not be summed as they have been. A male human and male C elegans are not remotely equivalent.*

**Response 10:** We thank the reviewer for highlighting these unintentional errors. The **Supplementary Material** has been revised accordingly: *“Whilst the search strategy was not limited to English, only articles translated into English were included.”* The mislabelling in **Table 1** has also been corrected accordingly: *“women/men”* has been changed to *“female/male”. C. elegans *are also now referred to separately as *“hermaphroditic/male”*.

**Comment 11:** *As ‘petite colonies’ only applies to yeast, I’m not sure separating it into three levels is warranted in this synthesis. All endpoints that appear in one organism could probably be collapsed into an ‘other’ or ‘model specific’ category and detailed individually.*

**Response 11:** We thank the reviewer for this comment and have made the suggested changes to **Table 1**. The subdivided categories for have been removed, with endpoints revised and listed under into: *“Model Specific Endpoints”*.

**Comment 12:** *There are serious issues with the ‘bias’ assessment portion of this manuscript. I don’t feel the ‘risk of bias’ application is clearly detailed or (without very clear details regarding how the Cochrane Risk of Bias tool was ‘adapted’) even reasonable for the types of articles reported. It is also completely unclear how a combined risk of bias can be included for all manuscripts on a given model – for example, there is one risk score for interassay variability for all human data (marked as ‘low’). This is not reasonable. The review would be better off without any ‘risk of bias’ assessment than with one that seems so highly at risk for bias itself. The authors also score everything as low or unclear. It is unclear what value the bias assessment has if everything scores the same way. How are mice and iPSC’s determined to be ‘low’ risk of selective reporting bias? I don’t see how that conclusion can be drawn. How is everything else unknown?*

**Response 12:** We thank the reviewer for this comment, with our changes detailed in **Response 1** sufficient to explain the methodology in the Risk of Bias assessment.

**Comment 13:** *Why is C. elegans described in the text as ‘Nematoda’? The entire phylum of Nematoda is not used as a model.*

**Response 13:** We thank the reviewer for highlighting this error, with all erroneous use of terminology corrected throughout.

**Comment 14:** *The ‘Molecular Recapitulation’ section should simply focus on 1) does the genetic manipulation / model accurately represent the genetic lesions that occur in humans and 2) do the molecular consequences of the manipulation/model accurately represent those occurring in humans? The authors include discussion of lower mtDNA in aging worms in this section, while they’ve eliminated the POLG mutator mouse studies from this review because they ‘model aging’. These are incompatible decisions.* *Some remarks should be provided regarding quality of assays – for example, standard qPCR is no longer a good option if quantitative data regarding mtDNA copy numbers is desired.* *“Generally causing cell cycle arrest at the L2 and L3 larval stages due to insufficient mtDNA levels.” This is a model, there is no evidence that convincingly demonstrates that it is a lack of mtDNA per se that leads to arrest.*

**Response 14:** We thank the reviewer for their concern and have amended the manuscript to reflect this. To ensure the clarity of this is conveyed in our review, the **Results** section has been amended: *“Finally, in polg mutant nematodes, cell cycle arrest was evidenced at L2 and L3 larval stages. In these worms, mtDNA content was consistently decreased compared to controls, though the mutations used are entire exon removals, in contrast to clinically reported POLG variants.”* As previously mentioned, the poor clinical recapitulation of *POLG*-related disease phenotypes in  the ‘mutator’ mouse is well known, while in nematodes this is unclear. This is further substantiated by the limited number of articles using *C. elegans* as a model of *polg-related research.* We also thank the reviewer for their comment on quality of assays. Assay methodology is outside the scope, and we refer readers/reviewer to relevant narrative reviews  [e.g. PMID: 32004343].

**Comment 15:** *What is the proposed mechanism for Clofilium tosylate? If a mechanism is not discussed, the drug should not even be mentioned.*

**Response 15: ** We have amended **paragraph 5** of the **‘Phenotypic Recapitulation’ Results** section to read *“* *using clofilium tosylate (CLO), an anti-arrhythmic agent targeting K+ ion channels ^24^,* *”*, providing a brief insight into the recognised action of CLO, with an added reference.

**Comment 16:** *Why would iPSC’s be considered a ‘gold standard’ over in vivo mouse models? I don’t think this is a view that anyone studying disease pathogenesis would agree with. Molecular features and cell autonomous features may be most directly modelled in iPSC’s, but features of disease pathology in tissue and in multi-system disease cannot even be approached. An ability to alter mtDNA levels in iPSC’s may not extend to in vivo relevance.*

**Response 16:** We would again like to thank the reviewer for this comment regarding iPSCs, which we have previously addressed in **Response 2**.

**Comment 17:** *The *“ *Sirt1 (mitophagy) pathway” is not a discrete/clear thing. Details need to be provided.*

**Response 17**: To clarify the findings of this article, the sentence has been reworded from *“related to POLG variants, chiefly through SIRT1-related mitophagy” to “related to POLG variants, via changes in metabolic signal transduction and increased mitophagic turnover ^13,18^.” *A reference to a review of SIRT1 and mitochondria is also provided (see PMID: 26831453).   We greatly appreciate the reviewer’s thoughtful feedback and have carefully implemented revisions to enhance the clarity, accuracy, and rigour of our review.
